# Radical cystectomy or trimodality therapy for muscle-invasive bladder cancer: a qualitative study exploring patient priorities and counselling needs when making a treatment choice

**DOI:** 10.1186/s12885-024-11927-1

**Published:** 2024-01-31

**Authors:** Flor Verghote, Charles Van Praet, Camille Berquin, Nicolaas Lumen, Karel Decaestecker, Ben Vanneste, Elke Rammant, Valérie Fonteyne

**Affiliations:** 1 Department of Radiation Oncology, Ghent University Hospital, Corneel Heymanslaan 10, Ghent, 9000 Belgium; 2 Department of Human Structure and Repair, Ghent University, Corneel Heymanslaan 10, Ghent, 9000 Belgium; 3 Department of Urology, ERN eUROGEN accredited centre, Ghent University Hospital, Corneel Heymanslaan 10, Ghent, 9000 Belgium; 4 Department of Urology, AZ Maria Middelares Hospital, Buitenring-Sint-Denijs 30, Ghent, 9000 Belgium

**Keywords:** Cystectomy, Muscle-invasive bladder cancer, Patient decision aid, Patient preference, Qualitative research, Quality of life, Radiotherapy, Shared decision-making, Trimodality therapy, Urothelial carcinoma

## Abstract

**Background:**

This study aims to explore the priorities and counselling needs of patients with muscle-invasive bladder cancer faced with a decision between radical cystectomy and trimodality therapy.

**Methods:**

We performed a qualitative study according to the phenomenological approach. Sixteen muscle-invasive bladder cancer survivors who underwent radical cystectomy or trimodality therapy completed a semi-structured interview between May 2022 and February 2023. Patients were recruited via Ghent University Hospital and a patient organisation. Data were analysed with inductive thematic analysis by a multi-disciplinary team using an iterative approach and investigators’ triangulation.

**Results:**

Four main priorities determining the treatment decision were identified. (1) curing the disease; (2) health-related quality of life (physical, mental and social); (3) confidence in the treatment, which was mainly based on trust in the clinician; and (4) personal attributes. Trust in the clinician can be achieved by fulfilling the patient’s information needs (accurate, complete, clear, impartial, personalised, realistic, and transparent information), ensuring accessibility of the clinician, and creating a clear and personalised treatment plan, involving patients to the extend they desire. Many patients considered a patient decision aid as a valuable asset in this process.

**Conclusion:**

Priorities vary between patients with muscle-invasive bladder cancer. Identifying individual priorities and offering personalised information about them is crucial for ensuring trust in the clinician and confidence in the treatment. Use of a patient decision aid can be beneficial in this process.

**Supplementary Information:**

The online version contains supplementary material available at 10.1186/s12885-024-11927-1.

## Background

Recommended treatments for patients with non-metastatic muscle-invasive bladder cancer (MIBC) are radical cystectomy (RC) and trimodality therapy (TMT). While both strategies have shown similar survival outcomes in selected patients, they have an unique set of advantages and disadvantages [1–4]. RC is a surgical procedure in which the urinary bladder, prostate, seminal vesicles and pelvic lymph nodes are removed, and a urinary diversion is created. Afterwards, patients often report lower health-related quality of life (HRQoL), including a decrease in urinary and sexual function (e.g. urinary incontinence and impotence). This also includes discomforts inherent to the constructed urinary diversion [5]. TMT is a bladder-sparing procedure, which consists of external radiotherapy of the bladder with a radiosensitizer, such as chemotherapy or carbogen and nicotinamide, after maximal transurethral resection of the tumour. This treatment involves several weeks of daily treatment and can be associated with urinary and gastrointestinal side effects, such as an overactive bladder or bowel and painful urination [6]. Afterwards, regular follow up with cystoscopy procedures is required. Salvage cystectomy will be necessary in about 15% of patients [1]. In patients eligible for both treatments, the treatment decision should be made through shared decision-making [7, 8]. However, informational support has been reported as an important unmet need in patients with bladder cancer [9]. Moreover, a recent qualitative study showed that uncertainties about treatment decisions could be limited if patients receive sufficient information about the different treatment options available [10]. To guide health care providers and patients in this process of shared decision-making, patient decision aids (PDA) are developed. An important step in PDA development is assessing the patients’ views on decisional needs [11]. To our knowledge there are no published qualitative studies focusing on the patient’s perspective regarding the treatment decision between RC and TMT. Therefore, this study aims to explore the priorities and counselling needs of patients with bladder cancer faced with a treatment choice between RC and TMT.

## Methods

### Study design

A qualitative study, according to the phenomenology approach, was conducted between May 2022 and February 2023 at Ghent University Hospital. The study protocol was approved by the Ethical committee of Ghent University Hospital (ONZ-2022-0037) on April 6, 2022. A signed informed consent was obtained from all participants. Qualitative data were reported following consolidation criteria for reporting qualitative research (COREQ) (Supplementary material 1).

### Patient sampling

Patients were eligible if aged 18 years or older, diagnosed with non-metastatic MIBC, underwent RC or TMT within 5-years before the interview and had no other primary tumour diagnosed less than 5 years before their MIBC diagnosis for which treatment was still required. Eligible patient treated at Ghent University Hospital were contacted by phone by their treating urologist or radiation-oncologist. Also, a recruitment call was made via Neovida, a Flemish bladder cancer patients association. Patients interested in participating were contacted by phone by the investigators. The reasons for conducting the research and interview process were explained. Purposive sampling with type of local treatment (RC and TMT), urinary diversion type (ileal conduit urostomy and neobladder), sex (i.e. as assigned at birth), and age was used to obtain maximum variation in these characteristics and to deepen emerging topics. Also, patients who were presented an equal choice between RC and TMT were favoured during sampling. Specifically, for patients recruited from our tertiary hospital, this determination was made through review of the physician’s notes within the patient’s electronic health record.

### Data collection

Interviews were performed at Ghent University Hospital or online via Microsoft Teams™ or Skype™, based on patient preference. The interviews were conducted in Dutch following a semi-structured interview guide designed by the radiation-oncology and urology department. Open-ended questions about two main topics were asked: (1) patient priorities that influenced the treatment decision, (2) counselling needs during the treatment decision process, including the potential role of a PDA. Patients were informed about what PDAs are before discussing their potential role in the MIBC treatment decision. The information given was based on the International Patient Decision Aid Standards (IPDAS) Collaboration definition [12]. Demographic, clinical and social characteristics of each patient were collected during the interview. Interviews were conducted by researchers (FV [MD, radiation-oncologist in training; male] or ER [post-doctoral researcher; female]) with experience in bladder cancer research. During each interview field-notes, capturing emotional reactions and behaviour, were recorded by the interviewer. Although, in some interviews the patients’ partner was present, only patient-driven-data was used for further analyses. Three participants had a prior established relationship with interviewer FV because of prior participation in another study (NCT04724928).

### Data analysis

All interviews were audio-recorded and transcribed verbatim. The interview transcripts were imported in NVivo™ (QRS International) for inductive thematic analysis. Thematic analysis was performed in accordance with the guidelines established by Baun and Clarke [13]. In an iterative process, a multidisciplinary team (VF [radiation-oncologist; PhD, MD; female], CVP [urologist; PhD, MD; male], MVDE [clinical nurse specialist; female], ER [post-doctoral researcher specialised in qualitative research; female] and FV [radiation-oncologist in training; MD; male] analysed the interview transcripts. Insights from researchers with different expertise ensured variability in data interpretation. Investigator triangulation was used to extend depth and ensure credibility of the data. During this process data relevant to the study aim was identified, of which a set of codes was generated. These codes were used to identify common themes and subthemes within the data. Themes were reviewed and revised by the broader research team. By matching the themes coherently, a thematic map was developed. The iterative process of data collection and analysis continued until data saturation was reached, meaning that no new insights emerged for our research questions.

## Results

In total, 16 interviews were conducted, lasting an average of 47 min (ranging from 29 to 74 min). Patient characteristics are presented in Table 1. A broad range of patient priorities (Topic 1) and counselling needs, including the potential role of a PDA (Topic 2), were identified.

**Table 1:** Characteristics of the patients

Demographics	*N* = 16
Age at diagnosis (Median,[IQR]), years	70 [10]
Sex (n men)	10
Nationality	
-Belgian	14
-Dutch	2
Treatment	
Radical cystectomy Urinary diversion type	9
-Ileal conduit urostomy	4
-Neobladder	5
Trimodality treatment	7
Months since treatment (Median,[IQR]), months	13 [25]
Additional therapies	
Neo-adjuvant chemotherapy	7
Adjuvant radiotherapy	0
Salvage cystectomy	1
Social status	
Having a partner (n yes)	11
Having children < 18y (n yes)	2
Work (n retired)	12

Abbreviations: IQR: interquartile range

## Topic 1: Patient priorities

Identified patient priorities were bundled in 4 main themes (i.e. curing the disease, health-related quality of life, confidence in the treatment, and personal attributes) and several subthemes. Table 2 provides an overview of these themes with accompanying patient quotes (Q) for illustration purposes. Indication of the context in which the quote was stated (i.e. pro or contra a specific treatment), is specified if applicable.

**Table 2:** Patient priorities influencing the treatment choice

Theme	Subtheme		Quote (Q)*
**Curing the disease**	Treatment characteristics	-Invasiveness	Q1: I did not choose for radiotherapy because there is a slightly higher chance of relapse. If there are no metastases and the bladder is removed you still have a decent chance of getting rid of it. *(Male, 70y, RC-urostomy)* Q2: I would rather go for a somewhat more extensive treatment that definitely everything is dead. *(Female, 48y, RC-neobladder)*
		-Timing	Q3: At the time I thought, the longer it’s there, the more chance you get metastases. You are consumed by fear. *(Male, 48y, RC-urostomy)*
	Disease characteristics		Q4: Had the tumour not been so large and had I had the certainty that it was not yet so far into the bladder wall, this treatment would certainly have been an option for me. *(Male, 48y, RC-urostomy)*
**Health- related quality of life**	Treatment characteristics	-Duration	Q5: I would not choose that trial and error process with 20 treatments. Then I would rather opt for the brief pain followed by a 4-day admission and then a rehabilitation period. *(Male, 67y, RC-neobladder)*
		-Treatment setting (In- vs. outpatient)	Q6: Not having to be hospitalised for so long helped in making my decision. *(Male, 65y, TMT)*
	Recovery period	-Duration	Q7: With a neobladder, your body needs several months to learn to accept it and not reject it. M*ale, 75y, TMT)*
		-Active involvement	Q8: A neobladder is at least a year of training, therefore I would prefer a urostomy. After the treatment you are already depressed and then this comes on top of it. *(Male, 70y, TMT)*
	Physical health	-Activity level	Q9: I want to be able to do all my work well. That’s because we’re living on a farm. Doing my house chores and cleaning, a little bit of everything. (Female, 79y, TMT) Q10: The ease of being able to go anywhere without worrying is the urostomy going to leak. Can I go swimming or not. Those kind of things. *(Male, 65y, TMT)* Q11: With a urostomy bag I can do everything. I can do everything like before. *(Male, 70y, RC-urostomy)*
		-Discomfort (pain, sleep, bladder function, urinary diversion,…)	Q12: I did not want that clock peeing. Because then you really have to set your alarm clock a few times at night to get up. I didn’t like that, I just want to have a good night’s sleep. (M*ale, 70y, RC-urostomy)* Q13: I am already a difficult sleeper and because of the bag you might not be able to twist and turn in bed the way you want. *(Female, 74y, TMT)* Q14: In case of bladder-preservation, you still have an effect on the bladder itself. The bladder shrinks. You get a kind of shrivelling anyway. *(Male, 70y, RC-urostomy)*
	Mental health	-Physical integrity vs. tumour removal	Q15: I preferred surgery because I wanted that filthy thing out of my body. *(Female, 48y, RC-neobladder)* Q16: The tumour had to go away. And I think radiotherapy could not give me that guarantee soon enough. *(Female, 48y, RC-neobladder)* Q17: An operation remains an operation. This was not an intervention. There is no cutting or slicing. *(Female, 84y, TMT)* Q18: You only feel half human when your bladder is taken away. *(Male, 65y, TMT)* Q19: I went for a neobladder because you are starting with something totally new. You are starting with a totally new organ. *(Male, 67y, RC-neobladder)*
		-Disease confrontation	Q20: With a stoma, you are confronted day and night. Always nurses. There’s always something. If you can just go to the toilet and help yourself, of course, that’s a big difference. *(Female, 79y, TMT)*
	Social health	-Social image	Q21: I don’t want to walk with a sign around my chest saying I am a cancer patient. I don’t want pity. I am still the same as before. *(Female, 74y, TMT)*
		-Participation social/professional activities	Q22: I thought the impact on work and family was important. I’m still young, so I have a lot of years to work. I also have a young family. *(Female, 32y, RC-neobladder)*
	Sexual functioning		Q23: I didn’t want to be disabled, no libido anymore. No woman is going to stay with someone like that, which is normal too. *(Male, 70y, TMT)*
**Confidence in the treatment**	Trust in clinician		Q24: At some point, the patient is completely in the hands of the medical profession in which the doctor makes decisions through his experiences in which he says this patient falls under this process and this one under this procedure. I would undergo both treatments though. *(Male, 67y, RC-neobladder)*
	Medical experiences	Own experiences	Q25: I have found on several occasions if something has to go wrong then it is with me. Something is always wrong. From surgery and hospitalisations, I have very bad memories. *(Female, 74y, TMT)*
		Experiences of others	Q26: I went to speak to someone who had undergone that operation. He found that it was very successful and after a few months he didn’t suffer from it anymore. I found that somewhat reassuring. *(Male, 48y, RC-urostomy)*
**Personal attributes**	Age/ Health status		Q27: I think it takes away a serious piece of your life, especially at my age. 65 year is pretty young, I believe. *(Male, 65y, TMT)*
	Sex		Q28: I still find a stoma different on a woman than on a man. I am quite a proud person. I can’t imagine that very well. *(Female, 74y, TMT)*
	Personality traits		

Abbreviations: TMT: trimodality therapy, RC: radical cystectomy

*Patient demographics (i.e. sex, age, and treatment) are specified in italic

### Curing the disease

Many patients stated that they wanted the treatment with the highest chance of curing the disease. More specifically, this meant the treatment with the highest chance of survival and/or the lowest chance of disease progression or relapse (Q1, contra TMT). Various factors influenced patients’ expectations of the success rates for different treatment options. Several patients linked the invasiveness of a treatment to its success rate, influencing their choice in favour of what they perceived as the more invasive treatment option (Q2, pro RC). Additionally, having a more advanced disease stage was mentioned as a factor favouring the choice of the more invasive treatment (Q4, contra TMT). Furthermore, due to fear for disease progression during the treatment free-period, several patients preferred the treatment that could be initiated the fastest. A few patients more specifically stated that they preferred the treatment that could physically remove the tumour from their body the fastest (Q3, pro RC).

### Health-related quality of life

The perceived impact of the different treatment options on the HRQoL played a prominent role in the majority of the patients treatment decision. Most patients were focused on the chance of making a ‘full’ recovery on the long-term. This was often formulated by patients as ‘the ability to regain an as normal life as possible’. Several patients placed particular importance on the usually more pronounced decrease in HRQoL during the rehabilitation period after treatment. For these patients, duration until anticipated recovery (Q7, contra RC-neobladder) and the degree of active involvement/ training required by the patient (Q8, contra RC-neobladder), were often mentioned as determining factors. Also, some patients treatment preference was influenced by the anticipated impact of specific treatment characteristics on their HRQoL during the treatment period. Some patients preferred the relatively short hospital stay associated with surgery (Q5, contra TMT), whereas others opted for the longer TMT treatment because of its outpatient setting (Q6, pro TMT). Other key determinants of HRQoL during the treatment period, that impacted the MIBC treatment decision, were identified. These determinants are described below for each HRQoL (sub)domain that was identified during this study.

### Physical health

#### Activity level

Patients often considered how the different treatment options would affect their daily activities, as this was an important determinant of their HRQoL. Patients indicated a wide range of specific activities that were important to them (e.g. physical exercises, gardening and shopping). In working patients, this also included professional activities (Q9). To maintain the desired activity level, patients frequently took into account the expected mobility decline in their choice of treatment. In particular, the construction of a urinary diversion was often expected by patients to reduce their mobility (Q10, contra RC-urostomy). In contrast, several patients with some degree of pre-existing bladder complaints saw a urinary diversion as an opportunity to regain some mobility (Q11, pro RC-urostomy).

#### Discomforts

The type and potential severity of discomforts after treatment also played an important role in patients’ decision process. A frequently expressed concern was regarding urinary discomforts, which were mainly treatment specific and often linked to sleep comfort. Several patients feared a RC because of potential urinary discomforts related to the constructed urinary diversion. A neobladder was sometimes seen as inconvenient because of the need to urinate at regular intervals, including at night, and the potential urinary incontinence and/or need of self-catheterization (Q12, contra RC-neobladder). In case of a urostomy, patients feared leakage, inflammation and sleep disturbance due to the presence of an external bag (Q13, contra RC-urostomy). In contrast, some patients preferred the surgical approach because TMT could impair their bladder function, leading to frequent and/or night-time peeing (Q14, contra TMT). A few patients also mentioned that the way a toilet visit would look after treatment, influenced their decision. One male patient indicated the importance of still being able to urinate standing up. Specifically concerning TMT, one patient indicated a fear of burns and nausea due to the radiation and chemotherapy, respectively.

### Mental health

Many patients indicated that their treatment decision was also driven by psychological factors. Some patients preferred surgery because they could not bear the presence of a tumoral mass in their body (Q15, pro RC). This often because of a fear for disease progression, mainly for metastasis (cf. curing the disease) (Q16, contra TMT). While others preferred a non-surgical option because it felt less invasive and would therefore better preserve the physical integrity of their bodies (Q17, contra RC). The decisive factor here was usually the ability to preserve the own natural bladder, which played an important role for their self-image (Q18, contra RC). In contrast, some patients specifically preferred a neobladder because they saw it as a fresh start (Q19, pro RC-neobladder). Furthermore, post-treatment care and visibility of the urinary diversion, mainly in the context of urostomy, were also indicated as negative because of continuous confrontation with the disease and its social impact (cf. social health) (Q20, contra RC-urostomy).

### Social health

Several patients expressed concerns about the visibility of the urostomy bag, fearing it might cause embarrassment or result in being perpetually labelled as ‘ill’ or as ‘cancer patient’ (Q21, contra RC-urostomy). Restriction of social activities due to dependence on care was also raised multiple times as an issue. This concern revolved primarily around the need for home nursing in the case of a urostomy. Also, the impact of the treatment on both family and professional life was considered by patients during the decision-making process (Q22).

### Sexual functioning

Several patients expressed concerns about impairment of their sexual functioning, citing issues such as erectile dysfunction, reduced libido, negative self-image, and being less attractive to the current or prospective partner (Q23, contra RC-urostomy). Sexual functioning was intricately intertwined with their physical, mental, and social well-being.

#### Confidence in the treatment

Patients often expressed the need to have confidence in the treatment before they are willing to undergo it. Often these patients’ confidence was largely based on the information provided by and the trust in their clinicians (mainly the physicians) (Q24). Other sources of information were also influential, although to a lesser extent (cf. patient counselling needs). In several cases, patients indicated that limited familiarity with a treatment option led to less confidence in it. However, personal medical experiences (Q25, contra RC) or experiences of peers (Q26, pro RC), whether negative or positive, can also influence patients confidence in a certain treatment. One patient expressed fear of general anaesthesia due to the perceived impact it had on her father’s mental health.

#### Personal attributes

Some patients cited personal attributes as influencing their treatment choice. For instance, age, specifically feeling young or old, and perception of their own overall health status, were factors some patients considered when opting for or against a particular treatment (Q27, contra RC). Often, these factors played a role in favouring or opposing what they perceived to be the more invasive treatment option. Additionally, one patient mentioned that sex and personality influenced the treatment choice (Q28, contra RC-urostomy).

## Topic 2: Patient counselling needs

During the interviews, patients expressed several needs regarding the counselling process, encompassing various information requirements, involvement in the construction of their treatment plan, and specific expectations from their clinicians (mainly of the physicians). Representative patient quotes regarding their counselling needs are provided in Table 3. Establishing trust in their clinicians emerged as a recurring theme, often tied to the fulfilment of their information needs. Patients stated the necessity for accurate, complete, clear, impartial, personalised, realistic, and transparent treatment information. Some patients indicated that they wanted a well-defined treatment plan, because it gave them a feeling of reassurance (Q29). However, patients had varying preferences for their level of involvement in the development of their treatment plan. While some patients expressed a desire to actively participate in creating this plan, others preferred a more passive role. In such cases, they conveyed their confidence in their clinicians’ ability to make the optimal treatment choice on their behalf (Table 2, Q24). Several patients highlighted the significance of having their priorities taken into account during the counselling process, considering it a crucial factor in feeling involved. Furthermore, patients emphasised the importance of clinician engagement and accessibility (Q30). Several patients mentioned that due to a lack of trust, they sought one or more second opinions of physicians, until this need was met. However, this process led in several patients to dissatisfaction and increased doubt (Q31). Outside their primary healthcare providers, some patients discussed treatment options with their family physician. However, the influence of family doctors on the final treatment decision was typically viewed as limited. Patients also sought insights from alternative information sources, often turning to family, friends, peers, and patient organisations for guidance (Table 2, Q26). Some patients turned to public sources, primarily the internet, to seek information (Q32). These sources included hospital websites, brochures, television, journal articles, and books. However, the impact of these sources on the final treatment decision was not always clear.

**Table 3:** Representative patient quotes (Q) regarding their counselling needs

Q29: I found it very important that reassurance was offered. That there was a way out of the situation I was in. (Male, 67y, RC-neobladder)
Q30: Some issues were given little thought. From the start of diagnosis to after treatment, I don’t think I saw the doctor for more than an hour. For a procedure with such a big impact on your life, that does not feel reassuring.*(Male, 48y, RC-urostomy)*
Q31: You better not ask for too many opinions. As a patient, that makes you doubt a lot more. *(Male, 48y, RC-urostomy)*
Q32: Via the internet, I started looking up several more things, such as treatment options in different countries. In the end, I still stuck to my decision. *(Male, 65y, TMT)*
Q33: When faced with such an illness, you are very much being lived and sometimes information passes you by. *(Female, 32y, RC-neobladder)*
Q34: It can help to think about the different treatment options and discuss them at home. That way you make a more conscious choice. *(Female, 32y, RC-neobladder)*
Q35: It has the advantage of not having a physician in front of you trying to shove just one solution down your throat, so to speak. *(Male, 48y, RC-urostomy)*
Q36: I find that if the physician has explained everything to you in detail that you don’t need that. It can only make you doubt. (F*emale, 79y, TMT)*
Q37: I wonder whether the information and solution that comes out of such a tool will apply to me and whether the information is correct. I think I would still find personal contact more reliable than a tool. *(Male, 48y, RC-urostomy)*

Abbreviations: TMT: trimodality therapy, RC: radical cystectomy

Patient demographics (i.e. sex, age, and treatment) are specified in italic

### Potential role of a treatment patient decision aid

**Fig. 1:**
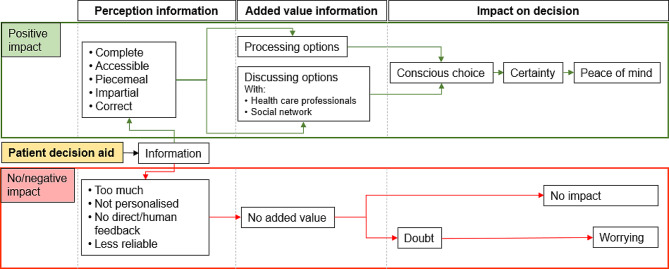
Patients’ perception regarding the role of a patient decision aid. Description: Schematic overview of the patients’ perception regarding the role of a patient decision aid in the decision making process between radical cystectomy and trimodality therapy. The green and red pathways indicate the potential positive or negative/absent impact of the patient decision aid, respectively

Many patients saw an added value in the use of a PDA, this primarily as a tool for obtaining, processing, and discussing information (Q34-35), all of which can be challenging following the diagnosis of MIBC (Q33). However, some patients had mixed or negative feelings about using a PDA (Q36-37). These differences in perception seem to be based on how patients perceive the additional information provided by this aid. Based on the conducted interviews a schematic overview of the patients perception regarding the role of a PDA in the decision making process between RC and TMT is presented in Fig. 1.

## Discussion

In this study, we identified several priorities and counselling needs of MIBC patients faced with a treatment decision between RC and TMT (Table 2). Four main priorities influencing the treatment decision were: (1) curing the disease, (2) health-related quality of life, (3) confidence in the treatment, and (4) personal attributes. Patients interpreted these priorities differently, attaching varying levels of importance and perceiving different impacts of the treatment options. These variations between patients led to differing treatment preferences. A qualitative study exploring treatment decision-making in patients with bladder cancer, without focusing on muscle-invasive disease or a particular treatment choice, similarly found that patients based their treatment decision on its effectiveness and compatibility with their lifestyle [14]. In our study, many patients viewed RC as the more effective treatment option, this due to its perceived invasiveness and faster tumour removal. This aligns with the results of a prior qualitative study among patients with prostate cancer. In this study, the surgical treatment option (i.e. robotic prostatectomy) was regarded by patients as a more effective treatment option when compared to external beam radiotherapy [15]. Importantly, none of these treatment modalities has demonstrated superiority over the other in terms of overall survival for prostate cancer [16]. Furthermore, in our study we observed that patients frequently opted for radiotherapy because they expected it to better preserve their body integrity and pre-treatment activity level. Similarly, patients with prostate cancer who chose for radiotherapy did so mainly because they believed it would have less impact on their body and lifestyle [15]. Additionally, elderly bladder cancer patients sometimes prefer what they perceive as the less invasive treatment option due to feelings of vulnerability [14]. Conversely, we have also observed that some younger patients declined what they consider the more invasive treatment, as they are currently in good health and do not want to compromise it. In contrast to our results, potential treatment side effects like incontinence, impotence, bowel dysfunction, and pain tended to hold a less significant position in the decision-making process of patients with prostate cancer [15]. Possibly, this can be attributed to more pronounced disparities in potential side effects between RC and TMT.

Further, patient were only committed to a particular treatment if they had confidence in it, with trust in their clinician being the paramount factor. As also observed in previous research, patients with bladder cancer often heavily rely on their clinician’s recommendations when making treatment decisions, sometimes without comprehensive understanding of the treatment and its consequences [14]. Similarly, in patients with prostate cancer, the decision between surgery and radiotherapy primarily depends on the physicians’ recommendations (mainly the urologist) [15, 17]. Also, patients with prostate cancer noted that both urologist and radiation oncologists tended to emphasize their respective specialties when providing information. The authors highlight the observed benefits of a combined clinic approach, where patients consult both a urologist and radiation oncologist, leading to a more balanced perspective on the treatment options [15].

Throughout the counselling process, clinicians should be aware of the difficulties patients face in absorbing pre-treatment information. Contributing factors to this challenge include limited basic knowledge about bladder cancer and the emotional stress associated with receiving a bladder cancer diagnosis [9]. In both our study and previous research, establishing trust in the information provider, tailoring communication to meet each patient’s unique needs, and providing ample time for patients to absorb information were identified as important supporting factors [9]. Addressing the impact of treatment on sexuality deserves particular attention, as several studies have specifically reported it as an unmet informational need in bladder cancer patients, especially in female and older patients [9]. If confidence in the treatment or trust in the clinicians is lacking, it may result in a frustrating process of seeking second opinions until this need is met, as was similarly described in a previous study [10]. Furthermore, previous research indicated that experiences of peers provide significant informational support for patients with bladder cancer when deciding on their choice of urinary diversion [10]. Similarly, we found that the confidence or reluctance of patients toward a specific treatment option can be strongly influenced by the experiences of their peers. Consequently, patient organisations can play an important role in the treatment decision-making process. However, we recommend that clinicians remain vigilant in ensuring that the information gathered from external sources is applicable to the patient’s unique situation.

Prior research has shown that PDAs can be of added value in the process of shared decision-making. The use of a PDA leads to improved knowledge, accurate perception of outcome probabilities, and reduced decisional conflict [18]. Our results suggest that a PDA could similarly enhance the decision-making process between RC and TMT (Fig. 1). However, to ensure its positive impact, it needs to be used as a supportive tool and may not replace patient-practitioner interaction, as also stated in previous research [19]. The results of this study can be used to develop or optimize/validate existing PDAs regarding the decision process between RC and TMT. Currently, a Dutch-language web-based PDA is available [20].

This study has several strengths and limitations. By purposive sampling and by recruiting patients via a tertiary hospital and a patient organisation we tried to obtain a broad view of the different patients’ priorities and counselling needs. However, all interviewed patients have Belgian or Dutch nationality. These countries have accessible and affordable healthcare systems, which may have resulted in the fact that logistical and financial incentives played less to no role in the treatment choice of most patients. Recruiting patients after the execution of the local treatment was considered the most feasible approach to achieve diversity in patient characteristics, particularly for less frequent characteristics. However, this retrospective approach introduces several important limitations. Firstly, the extent to which patients had a choice between RC and TMT is uncertain. Secondly, patients’ responses regarding their treatment priorities and counselling needs might be influenced by post-treatment experiences, such as treatment side effects. Recall bias was minimised by excluding patients treated more than 5 year prior to the interview. To mitigate confirmation bias in favour of any specific local treatment, we developed the interview guide using a multidisciplinary approach and employed investigator triangulation during thematic analysis. Additionally, the IPDAS definition guided the provision of objective information about PDAs to the patients.

As this is an exploratory study, it is recommended to conduct additional research involving patients before or during the decision-making process to validate the current findings. Furthermore, the impact of implementing PDAs in guiding local treatment choices for patients with MIBC should be further evaluated via prospective studies focusing on outcomes such as decision regret and treatment satisfaction.

## Conclusions

This study provides an overview of the various priorities and counselling needs of MIBC patients faced with a decision between RC and TMT. Priorities vary between patients with MIBC. Identifying the individual priorities and offering personalised information about them is crucial for ensuring trust in the clinician and confidence in the treatment. Use of a PDA can be beneficial in this process.

### Electronic supplementary material

Supplementary material

## Data Availability

The datasets generated during the current study are not publicly available due to the sensitive nature of the data and the ability to identify participant’s identity from the raw data. The data (in Dutch) may be available from the corresponding author on reasonable request.

## Abbreviations

COREQ: Consolidation criteria for reporting qualitative research
HQRoL: Health-related quality of life
IPDAS: International Patient Decision Aid Standards
MIBC: Muscle-invasive bladder cancer
RC: Radical cystectomy
TMT: Trimodality therapy
PDA: Patient decision aid
Q: Quote
